# Global Healthcare Systems: Perspectives on Pathology Practices in Bulgaria

**DOI:** 10.7759/cureus.41434

**Published:** 2023-07-06

**Authors:** George S Stoyanov

**Affiliations:** 1 Department of Pathology, Complex Oncology Center, Shumen, BGR

**Keywords:** healthcare access, healthcare funding, diagnosis, autopsy, pathology, bulgaria, global healthcare systems

## Abstract

Healthcare in Bulgaria is a universal and free right as per legislation. However, due to limited government funding from a singular state-run insurance fund and the inequities in the distribution of limited healthcare professionals, access to quality healthcare is difficult for most citizens. Pathology is one of the most severely affected specialties as it is not only regarded as one of the most difficult ones due to its aspects and difficulties in obtaining it but also due to the lack of funding. The healthcare fund does not reimburse immunohistochemistry or genetic tests; however, hospital reimbursement relies on pathological diagnosis in most cases, prompting peer and institutional pressure towards adapting the diagnosis. A relatively good aspect of all of these is the low cost of immunohistochemistry if the patient chooses to pay for such, albeit when compared to the mean monthly salary within the country, the cost is still high. Lack of funding is not only limited to biopsy diagnosis, with research funding being minimal at best, while autopsy practice receives no funding whatsoever.

## Editorial

The Bulgarian Healthcare system is in a dubious position, from its point of legislation to its everyday implementation in different types of healthcare facilities. By law, universal healthcare is free for all citizens and subsidized by a single state fund - National Health Insurance Fund (NHIF) [1]. The budget of the NHIF is formed by individual payments from working citizens and direct government funding [1]. The personal payments from working citizens are equivalent to 8% of the individual monthly salary (separated into employee and employer branch payments), with the minimal income to be withdrawn from being 355 Bulgarian Leva (BGN), equivalent to roughly 181 Euro or 199 United States Dollars (USD); on the other hand, the maximum income to be withdrawn from is 3,400 GBN equivalent to 1,737 Euro/1,903 USD [2,3]. Therefore, the healthcare tax for working individuals varies from 28.4 BGN (14.5 Euro/15.9 USD) to 272 BGN (139 Euro/152 USD). For children under 18, pensioners (for age, working experience, social and medical reasons), and prisoners, the state deposits a monthly 28 BGN (14.3 Euro/15.7 USD), with the same fee applying for self-ensuring non-working individuals. Individuals under direct government employment are free from healthcare tax [4].

Additionally, other than the individual healthcare tax, each year, as part of the state budget and the budget of the Ministry of Healthcare, the state deposits a percentage of the gross domestic product (GDP) to the NHIF, generally around 2-4%, to equal a total of 6 Billion BGN (3.07 Billion Euro/3.36 Billion USD) as the total budget [5]. As per the formulation of the healthcare laws, this is separated into different budget branches for prevention, treatment, medication (including partial or full reimbursement for medications for home treatment for socially significant diseases), follow-up, and rehabilitation of the population, which is equivalent to roughly 6.6 million citizens as per the 2021 census. This equals, at best, a total of 1,000 BGN (511.6 Euro/588.9 USD) as an individual annual healthcare sum for each citizen [5]. Furthermore, part of these funds go towards dental healthcare, a maximum of around 170 BGN (87.3 Euro/95.5 USD) per individual per year, with the remained of needed dental healthcare covered by the individual at prices set by nearly 7,500 dental medics within the country [6].

On its own, this sum is grossly insufficient to cover the healthcare bills of the population, but when it comes into account that part of this sum is spent not only to cover the cost of treatment but also for healthcare institutions to pay their bills (electricity, water, heating, etc.) and is the basis of formation of the health workers pay it becomes evident that this budget is insufficient to cover the costs of healthcare. From this gross underfunding comes the first contradiction between the healthcare laws and formulations and how they are interpreted, as individuals that do not pay their healthcare tax must pay all expenses for treatment, as per the individual prices set by the healthcare facilities.

Furthermore, it becomes evident, even without a detailed analysis, that not only is the budget of the NHIF insufficient to provide free healthcare to the more than 6 million citizens, but also that the limited number of medical professionals (a little less than 30,000 in the country) is grossly overworked and underpaid, with a mean salary of 668 to 3,015 BGN depending on the region, level of healthcare and specialty (342-1,542 Euro/374-1,688 USD) [6].

For comparison, the average GDP percentage for healthcare in the European Union is 8%, and in states with a significantly higher GDP and other than state funds, there are multiple privately run healthcare insurance groups with a considerably higher monthly healthcare tax/fee.

Despite free healthcare being a fundamental right of all citizens, the NHIF covers only some "basic" steps in the diagnostic and treatment process, with patients having to pay for additional procedures and materials not mentioned in the "clinical pathway" for which they are treated, e.g., laparotomy for colorectal carcinoma is fully covered. Still, if the patients choose or are advised by the surgeon to undergo a laparoscopic or robot-assisted procedure, they must cover the total cost.

As all hospitals in the country, regardless of them being private, municipal, or state-owned, are registered as joint stock companies, they are not only free to place their pricing for additional payments, including preferential treatments but also to keep a significant sum of what the patient pays and transferring minimal amounts to the treating physician. Furthermore, some institutions outright force the patients to cover the cost of treatments; as such, they receive more money than the state would otherwise transfer for therapy.

A limited number of hospitals receive further state investments that typically cover the renovation of facilities and new costly equipment, research investments, and others, which rarely reach their desired goal. Privately owned hospitals also have the opportunity to be reimbursed by the NHIF; however, they rarely choose to operate in this manner. While they typically offer higher quality healthcare, they are also in several hubs. At the same time, a lot of districts have none or a few small privately owned hospitals with limited capabilities.

Pathology practice in Bulgaria

One of the most significantly affected specialties by these significant flaws in the system is pathology (surgical pathology, general and clinical pathology, pathological anatomy). As it is widely accepted as one of the most challenging specialties in medicine, from the roughly 30,000 medical doctors in Bulgaria, only around 100 are practicing pathologists, with residents in training and consulting pensioners elevating the number to no more than 140 pathologists (0.47% of practicing physicians are pathologists compared to 5.13% being general surgeons and 5.85% being gynecologists) [6]. Furthermore, not all districts have the same density in the distribution of pathologists per capita, with districts in which university centers are present having roughly 10-25 pathologists. In contrast, most districts have only one or two, with several districts relying on traveling pathologists from neighboring or distant districts to cover for histopathological diagnosis.

Despite the lack of pathologists, monthly salaries remain low, often lower than physicians' average salary, and most pathologists have to work in two or more healthcare facilities. This is coupled with pathology being a singular specialty, not separated into topographical fields, such as gastrointestinal, neuro, and gynecologic pathology, as in most countries. It is not unusual for a single pathologist to process over 1,000 biopsy specimens (histological and cytological) at their primary institution and 500 at their secondary annually, with more than 5,000 histological slides.

Despite a significant number of procedures funded by the NHIF requiring histopathological confirmation, sometimes relying on a single word to cover the funding, e.g., endometrial polyp as a diagnosis is non-acceptable while adenomatous endometrial polyp is the one accepted, funding for histopathology is limited. Furthermore, in almost all cases when the biopsy material is insufficient or inadequate for diagnosis, e.g., superficial gastric mucosa, debris, or necrotic tissue only present in the specimen, if the biopsy is interpreted as undiagnostic or insufficient, the NHIF will not reimburse the hospital stay and treatment, prompting peer and institutional pressure towards the pathologist to imagine, adapt, or overdiagnose inadequate specimens (prompting workaround diagnosis such as superficial gastritis, specimens from gastric ulcer base, granulomatous tissue from abscess wall, etc.).

The only funding for immunohistochemical testing is carried out for breast malignancies (only for hormonal receptor, proliferative index, and target treatment), while for all other locations and diseases, the cost of testing is covered on goodwill either by the institution (in an exceedingly limited manner, only for otherwise costly procedures) or by the patients themselves. The NHIF does not cover genetic testing and predictors for treatment and response.

This often limits and makes the pathological diagnosis impossible at the same time that NHIF reimbursement relies on it. Hence in cases where the nosological unit cannot be identified without immunohistochemistry, terms such as malignant blastoma are pretty common, as well as unofficial workaround terms such as diffuse astrocytic glioma with a morphology akin to glioblastoma (when immunohistochemical and genetic tests have not been performed, but the NHIF request the presence of glioblastoma in the pathological diagnosis).

One relatively good aspect of this is that in the few centers where immunohistochemistry is available, the cost is exceedingly low compared to the price in more developed countries. At the same time, there are also many commercial (company-funded) and private (charity-funded) programs to cover the cost of such additional tests, but only for treatment-response-related markers. The average price of a single antibody tested, including all necessary materials, ranges from 80 to 120 BGN depending on the antibody (40.9-61.4 Euro/44.7-67 USD), and a single consultation fee for the pathologist handling the case ranging from 50 to 100 BGN (25.6-51.2 Euro/28-55.9 USD). As such, while the overall cost for three to four antibodies tested may seem low for developed countries, a patient with a malignancy of unknown primary or rare malignancy often has to pay more than half of an average monthly salary for the diagnosis to be defined and treatment to initiate.

Aside from biopsy specimens, as in all other regions, an autopsy is also a fundamental workload of pathologists in Bulgaria. While in the previous decades, autopsy rates exceeded 95% of all deceased patients, currently autopsy halls are significantly rundown, as most of them have not undergone renovation since their construction (typically constructed in the period 1950-1985) or for those limited who have undergone renovation, those have been predominantly cosmetical, most autopsy halls do not cover the basic requirements for modern-day autopsy and autopsy rates are significantly decreased. Partly due to this, only a handful of institutions cover the generally accepted 10% autopsy rate of diseased patients, assuring some quality control for treatment; most institutions perform a limited number of autopsies (typically ranging from 10 to 40), and many healthcare institutions, predominantly those without full-time pathologists, do not perform autopsies at all. In cases when an autopsy is requested, typically, the diseased is transferred to another healthcare facility for such to be performed. The second and foremost reason for the limited and further decreasing number of autopsies is the outright denial of institutions and pathologists to perform autopsies, as the NHIF does not cover the cost of such. Neither the pathologist nor hall attendants receive any pay, while the healthcare facility loses money for formaldehyde, paraffine embedding, slide preparation, and staining.

Scientific research

Due to the legislation on university staff and teachers, all assistant professors must obtain a Ph.D. thesis within four years of being hired. As most centers where a specialty can be obtained are university centers, a newly graduated resident must multitask even more than so described [7]. In these four years, he must get basic pathological literacy in all fields of pathology so that he can grossly process biopsies from varying systems on his own and place a histopathological diagnosis, have seminars with students (usually between five and eight seminar weekly, spanning one and a half hours each), perform autopsies and detailed scientific work on a specific topic, in which he usually has no prior knowledge and experience. Financing this research is also very challenging, with a state scholarship of 500-1,000 BGN (255.42-510.84 Euro/250.38-560.77 USD) monthly for the duration of the thesis research and additionally a single project fund of 7,000 BGN (3,575.86 Euro/3,925.37 USD), of which some of the money is reserved for reviewers of the project and institutional and government fees. The structuring of these laws has several effects, as burnout is high due to the overload, whit many simply having to resort to "phoning it in" in many of the mentioned work-related engagements. The quality of scientific research also suffers from this, as with these levels of subsidy, the materials (machinery, reagents, software, etc.) that can be obtained are limited in their number and novelty, and even a small article processing charge to publish the finalized work is impossible to pay, with the few good results being published in national journals, typically by the institution in which the trainee works and are grossly unavailable to the general scientific public.

Is there a light in the dark

As seen by the data depicted so far, not only pathologists but medical doctors, in general, are overworked and underpaid. The significant difference in the density of physicians per capita makes healthcare access difficult for the population and the workflow uneven. While the current healthcare legislation, introduced and first implemented in the early 2000s, has its benefits as it basically allows for free access and relatively cheap access to good quality healthcare for most developed countries, the budgetary aspects have not been adjusted for since first introduced. Therefore significant changes are required, either in adjustment to the healthcare tax - the removal of the maximum limit to be withdrawn from (in this instance, someone with a salary of 10,000 BGN (5,108.37 Euro/5607.68 USD) would pay 800 BGN (408.67 Euro/448.61 USD) healthcare tax instead of the maximum fixed 272 BGN, adjustment of government payments for the groups exempt from tax (from 28 BGN, which is below the minimum individual tax to the mean collective healthcare tax payments of individuals, for example to 50 BGN - these alone would more than double the healthcare budget of the state) and the introduction of additional private, collective or specialized healthcare funds would drastically increase the healthcare budget of the state and allow for more subsidizing, an expanded spectrum of covered materials and tests, higher wages and would limit the drain of newly graduated, as well as experienced doctor abroad, with the probability of louring foreign medical specialists to the country. Increased GDP influx to the mean European values would also greatly benefit the renovation and restoration of healthcare facilities, improving healthcare access in multiple districts.

Additionally, residents should be allowed to focus on their pathological training (this also applies to all fields and specialties) and not be forced to have excessive student seminars and perform scientific research without prior experience, especially with the laughably small scholarship and budget for research. After completing their training, these individuals will be more adept at performing these tasks based on their experience. They will arguably have more time to do so and a lesser chance of burnout. Government and additional subsidizing of scientific work should also, of course, be significantly increased in parallel to the increase of the healthcare budget.

Admittedly from all aspects, not only pathology in Bulgaria but the whole healthcare system is more than 50 years behind that of developed countries, of which we are striving to be one and in dire need of significant reforms. Sadly these reforms, which do not need to be built from the ground up, but adapted and introduced from better-functioning healthcare systems, are never talked about and omitted from the political and public spectrum, mentioned briefly when a sensation needs to be created (one of the most significant talking points for the past four decades has been the construction of a National Pediatric Hospital, one of which there already is, but it is never mentioned). However, the few remaining Bulgarian healthcare workers and patients pay the bill for these omissions and mistakes.

Catch-22

From legislation to practice, there are a lot of contradictions and problems in Bulgarian healthcare. The lack of financing and personnel, the rundown facilities, and severe discrepancies in the distribution of healthcare access make it impossible for healthcare quality to be ubiquitous and equal to that of other European countries. Several reforms need to be implemented, which would improve some aspects of the situation. Regarding pathology, one of the benefits of the aforementioned facts is the low price of services compared to other more developed counties.

Lack of recognition, peer and institutional pressure, the difficulty of the unsegregated specially and low pay, and the machinations of obtaining a specialty are also discouraging obstacles towards newly graduated physicians choosing pathology as their specialty.
